# A Rare Case of Myelin Oligodendrocyte Glycoprotein Antibody-Associated Transverse Myelitis in a 40-Year-Old Patient With COVID-19

**DOI:** 10.7759/cureus.23877

**Published:** 2022-04-06

**Authors:** Sotirios G Doukas, Andrea P Santos, Waleed Mir, Sarosh Daud, Tracy H Zivin-Tutela

**Affiliations:** 1 Department of Forensic Sciences and Laboratory of Toxicology, University of Crete, School of Medicine, Heraklion, GRC; 2 Department of Medicine, Saint Peter’s University Hospital, New Brunswick, USA; 3 Department of Internal Medicine, Saint Peter’s University Hospital, New Brunswick, USA; 4 Department of Community Medicine, Shalamar Medical and Dental College, Lahore, PAK; 5 Department of Infectious Disease, Saint Peter’s University Hospital, New Brunswick, USA

**Keywords:** myelin oligodendrocyte glycoprotein (mog), sars-cov-2, myelin oligodendrocyte glycoprotein antibody, covid-19, acute transverse myelitis (atm)

## Abstract

The coronavirus disease 2019 (COVID-19) includes an extensive spectrum of clinical manifestations of severe acute respiratory syndrome coronavirus 2 (SARS-CoV-2) infection. Previous studies have shown that SARS-CoV-2 often exhibits central nervous system (CNS) manifestations, including encephalitis, meningitis, and spinal cord pathologies. To date, few cases of COVID-19-associated transverse myelitis (TM) have been described.

A 40-year-old unvaccinated man with no significant medical history presented to the emergency department complaining of fever, worsening burning sensation in his lower extremities, unsteady gait, and difficulty initiating urination for five days. Twelve days before presentation, the patient had tested positive for SARS-CoV-2 infection. Physical examination revealed hyperesthesia, starting around the nipple line (T4) and extending distally, involving the lower extremities, accompanied by symmetric weakness in the lower extremities. Magnetic resonance imaging of the thoracic spine with and without contrast revealed mild intramedullary signal abnormality at T3-T4 and T6-T8, confirming the suspicion of TM. Further laboratory testing revealed a C-reactive protein level of 67 mg/L, lactate dehydrogenase level of 181 mg/L, serum B12 level of 781 pg/mL, methylmalonic acid level of 165 nmol/L, folate of >24.5 ng/mL, and thyroid-stimulating hormone level of 0.481 μIU/L. Lumbar puncture was performed, and cerebrospinal fluid analysis revealed a cell count of 14 cells/µL, with 69% lymphocytes, glucose level of 81 mg/dL, protein level of 32 mg/dL, and negative cultures. Human immunodeficiency virus, antinuclear antibody screening, anti-DNA, rapid plasma reagin, Lyme serology, anti-SSA, and anti-SSB antibodies were unremarkable. Serum aquaporin-4 immunoglobulin G was negative, and myelin oligodendrocyte glycoprotein (MOG) antibodies were positive. The patient was treated with intravenous methylprednisolone and oral gabapentin and was discharged after five days when his urinary retention improved.

Most previously reported cases of COVID-19-related TM were negative for autoimmune workup. Although the exact pathophysiology of COVID-19-related TM remains unclear, one hypothesis suggests that it is a consequence of the direct viral invasion. However, our patient had MOG antibodies, suggesting the possible involvement of a different mechanism. In MOG-associated TM, it has been suggested that MOG antibodies gain access to the CNS through disruption of the blood-brain barrier.

This unique presentation demonstrates that further studies are needed to understand the effects of SARS-CoV-2 infection on the immune and nervous systems. It also highlights that young and otherwise healthy patients are at risk of severe COVID-19-related complications, including CNS disorders.

## Introduction

Coronavirus disease 2019 (COVID-19) includes an extensive spectrum of clinical manifestations of severe acute respiratory syndrome coronavirus 2 (SARS-CoV-2) infection [1]. The first reported case of SARS-CoV-2 infection was in Wuhan, China, in December 2019. Since then, the worldwide spread of SARS-CoV-2 has led to an increase in the number of COVID-19 cases at the pandemic level [1].

Although COVID-19 usually includes respiratory symptoms, such as cough and shortness of breath, non-respiratory manifestations have also been recognized [1]. Previous studies have shown that COVID-19 often exhibits central nervous system (CNS) manifestations, including encephalitis, meningitis, and spinal cord pathologies. To date, few COVID-19-associated transverse myelitis (TM) cases have been described in the literature [2]. Here, we present a case of COVID-19-induced TM with positive myelin oligodendrocyte glycoprotein (MOG) antibodies.

## Case presentation

**Figure 1:**
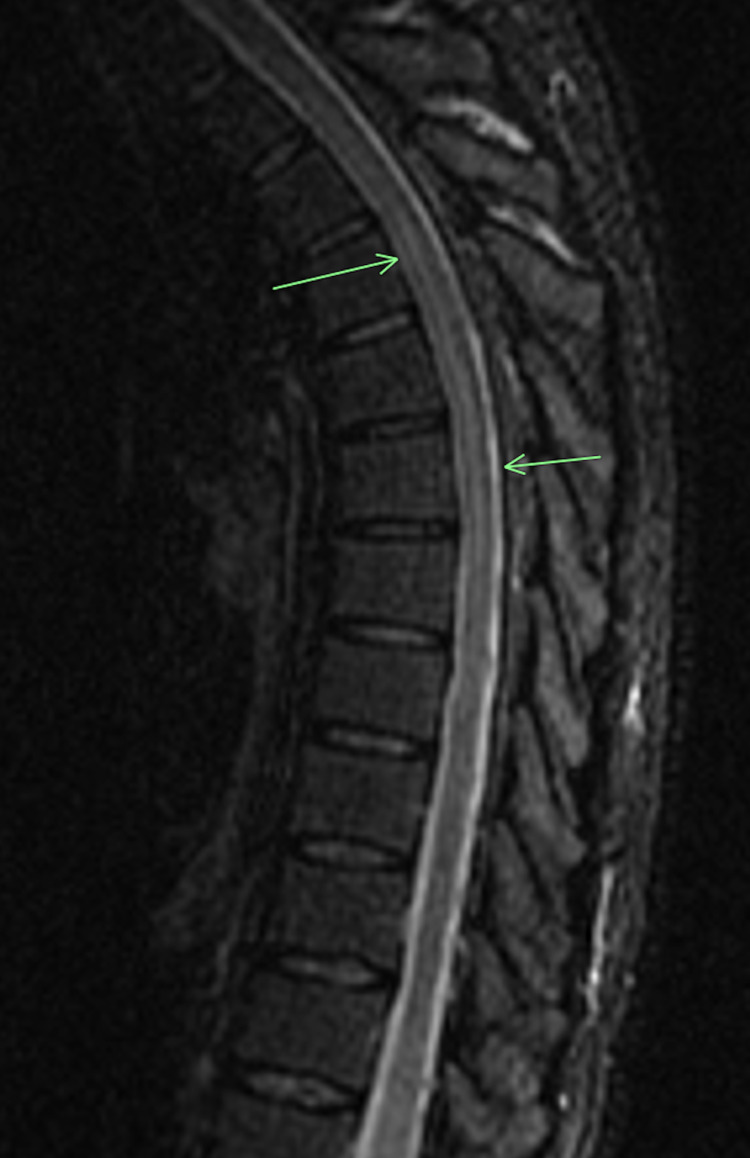
Short-tau inversion recovery MRI of the thoracic spine revealing mild intramedullary signal abnormality at T3-T4. MRI: magnetic resonance imaging

A 40-year-old unvaccinated man with no significant medical history presented to the emergency department (ED) complaining of fever, worsening burning sensation in the lower extremities, unsteady gait, and difficulty initiating urination for five days. The patient reported a previous ED visit due to difficulty voiding when straight urinary catheterization was performed, and tamsulosin was prescribed at discharge. However, given the worsening of his symptoms, he returned to the ED for re-evaluation. On presentation, he described a burning sensation in the lower back and abdomen, extending to his thighs. No trauma, rash, recent travel, or tick bite was reported. Twelve days before presentation, he had tested positive for SARS-CoV-2 (polymerase chain reaction (PCR) nasopharyngeal swab). On admission, his vital signs were as follows: temperature, 99°F; blood pressure, 119/78 mmHg; pulse, 74 beats per minute; respiratory rate, 17 breaths per minute; and oxygen saturation, 96% on room air. Physical examination revealed hyperesthesia, starting around the nipple line (T4 dermatome) and extending distally, involving the lower extremities, accompanied by symmetric weakness in the lower extremities. The cranial nerves were grossly intact, patellar reflexes were +3 bilaterally, and unsteady gait was noted. The initial inpatient laboratory workup showed white blood cells of 9,200 cells/mm^3^ with an absolute lymphocyte count of 1,000 cells/mm^3^ and platelet count of 438,000 cells/mm^3^. Chest radiography was performed because of the recent SARS-CoV-2 infection, which revealed focal and reticular opacities in the right lower lobe. Magnetic resonance imaging (MRI) of the thoracic spine with and without contrast revealed mild intramedullary signal abnormality at T3-T4 (Figure 1) and T6-T8, confirming the suspicion of TM. Following his imaging results, the medical team initiated the administration of intravenous methylprednisolone 1 g daily and oral gabapentin 100 mg twice daily. In addition, a Foley catheter was inserted for his persistent urinary retention.

Further laboratory testing revealed a C-reactive protein level of 67 mg/L, lactate dehydrogenase level of 181 mg/L, serum B12 level of 781 pg/mL, methylmalonic acid level of 165 nmol/L, folate of >24.5 ng/mL, and thyroid-stimulating hormone level of 0.481 μIU/L. A lumbar puncture was performed, and cerebrospinal fluid (CSF) analysis revealed a cell count of 14 cells/µL, with 69% lymphocytes, glucose level of 81 mg/dL, protein level of 32 mg/dL, and negative cultures. Human immunodeficiency virus, antinuclear antibody screening, anti-DNA, rapid plasma reagin, Lyme serology, anti-SSA, and anti-SSB antibodies were unremarkable. Serum aquaporin-4 immunoglobulin (IgG) was negative, and MOG antibodies were positive. His recent positive SARS-CoV-2 test results, clinical presentation, MRI, CSF, and laboratory findings further supported the diagnosis of acute MOG antibody TM. The patient responded appropriately to the course of steroids, and his symptoms gradually improved, with a resolution of hyperesthesia and improvement of gait. The patient eventually regained his bladder function after five days of treatment and was able to independently void. The patient was discharged on a tapered prednisone regimen for 18 days and advised to follow up with his primary doctor and a neurologist within a week.

## Discussion

Anti-MOG syndrome is an immune-mediated inflammatory condition of the CNS that frequently affects the optic nerves and spinal cord [3]. MOG is a membrane protein expressed on oligodendrocyte cell surfaces and the outermost surface of myelin sheaths, making it a target for autoimmune processes. In adults, anti-MOG syndrome most commonly presents with optic neuritis and myelitis [4]. Adult CNS demyelination associated with MOG antibodies is usually not associated with infection or vaccination. However, there are few case reports of infection and vaccination-associated demyelinating CNS syndromes [5,6].

Recently, COVID-19-related TM cases have been reported [7]. To mention a few, Zhao et al. reported the case of a 66-year-old man with acute myelitis after a SARS-CoV-2 infection [8]. However, no MRI or CSF analysis was performed, given the ongoing pandemic protocol, and MOG antibody testing was not performed either. Sarma et al. also presented a SARS-CoV-2-related TM case in a 28-year-old woman with MRI with and without contrast, showing extensive findings throughout the spinal cord [9]. Lumbar puncture showed mononuclear cells (125/μL; protein of 0.6 g/L, normal glucose, negative antibodies, and culture-negative for infection). Valiuddin et al. described COIVD-19-induced TM in a 61-year-old man that was confirmed with an electromyogram [10]. CSF analysis revealed elevated protein and albumin levels, with a white cell count of 1/mm^3^ and mature lymphocytes on cytology. The autoimmune encephalopathy panel and SARS-CoV-2 PCR assay results were negative in the study by Valiuddin et al.

Although several cases of TM have been described in the literature, MOG antibodies have been negative or not reported in most patients. Similar to our case, Dias da Costa et al. reported the case of a 31-year-old man with symptoms of myelopathy following a SARS-CoV-2 infection in which thoracic and lumbosacral spinal cord MRI identified longitudinal extensive TM. All systemic inflammatory, autoimmune, and infectious markers were unremarkable and non-specific; however, serum analysis showed a low positive titer of MOG antibodies [11]. Two other myelitis cases following SARS-CoV-2 infections associated with MOG antibodies have been reported. One case reported an unusual presentation of human herpesvirus 6 myelitis with concomitant high-titer MOG antibodies in COVID-19. Zhou et al. also described the case of a young man with bilateral optic neuritis with disc edema and longitudinally extensive TM who was simultaneously SARS-CoV-2-positive and MOG antibody-positive [12]. Overall, these cases of TM may have occurred simultaneously or after COVID-19, and the neurological symptoms included lower extremity weakness, sensory deficits, and bladder or bowel dysfunction in most cases.

Although the exact pathophysiology of COVID-19-related TM remains unclear, one hypothesis suggests that it is a consequence of the direct viral invasion. SARS-CoV-2 could invade oligodendrocytes in the CNS through the angiotensin-converting enzyme-2 receptor [2]. However, our patient had MOG antibodies, suggesting the possible involvement of a different mechanism. In MOG-associated TM, it has been suggested that MOG antibodies gain access to the CNS through disruption of the blood-brain barrier [13]. Once it accesses the CNS, it targets MOG expressed on oligodendrocytes, leading to antibody-mediated myelitis, encephalitis, or optic neuritis [14]. Despite this hypothesis, further clinical and pre-clinical investigations are necessary to reveal the underlying mechanism of COVID-19-related MOG TM.

## Conclusions

Here, we report a case of TM in a COVID-19-positive patient with MOG antibodies. This unique presentation demonstrates that further studies are required to understand the effects of SARS-CoV-2 infection on the immune and nervous systems. It also highlights that young and otherwise healthy patients are at risk of severe COVID-19-related complications, including CNS disorders.
